# A calcaneal osteochondroma with recurrence in a skeletally mature patient: a case report

**DOI:** 10.4076/1757-1626-2-7013

**Published:** 2009-06-23

**Authors:** Mustafa Koplay, Serdar Toker, Levent Sahin, Volkan Kilincoglu

**Affiliations:** 1 Department of Radiology, Medical Faculty, Dumlupinar University, Kutahya, Turkey; 2 Department of Orthopaedics and Traumatology, Medical Faculty, Dumlupinar University, Kutahya, Turkey; 3 Department of Anesthesia and Intensive Care, Medical Faculty, Dumlupinar University, Kutahya, Turkey

## Abstract

**Introduction:**

Osteochondroma is the most common benign tumor of the skeleton. However, calcaneal osteochondroma is very rare. Osteochondromas grow during childhood through adolescence, but usually growing ends when the epiphyseal plates close. In an adult, growth of an osteochondroma suggests the diagnosis of malignant transformation to a chondrosarcoma. However, enlargement of an osteochondroma reported as benign after skeletal maturity is present in literature.

**Case presentation:**

We report the clinical and radiologic findings of a calcaneal osteochondroma with an extremely rare placement and painfull, rapid reccurence following surgical excision in a skeletally mature female. The lesion showed growth the first-operation later and was re-operated. Histopathological examination did not show malignancy.

**Conclusion:**

It should kept in mind that benign osteochondromas can show symptomatic growth in skeletally mature patients without malignant transformation.

## Introduction

Osteochondroma is the most common benign tumor of the skeleton. It usually rises from the metaphyseal or metadiaphyseal region of long bones of the appendicular skeleton and are most commonly seen around the knee [1,2]. Osteochondromas grow during childhood through adolescence, but usually growing ends when the epiphyseal plates close [1,3]. They are typically described in patients younger than 20 year-old and extensive osteochondroma growth into adulthood is rarely reported [3,4]. In an adult, growth of an osteochondroma suggests the diagnosis of malignant transformation to a chondrosarcoma [3,5]. However, Krieg et al [6] and Nogier et al [3] reported extensive growth of an osteochondroma in a skeletally mature patient whose tumor had no evidence of malignancy in histologic examination. In the foot and ankle, osteochondromas are uncommon. Calcaneus is one of the most unusual region for an osteochondroma. In this study, we describe the clinical and radiologic findings of a calcaneal osteochondroma with an extremely rare placement and rapid reccurence following surgical excision in a skeletally mature female.

## Case presentation

**Figure 1:**
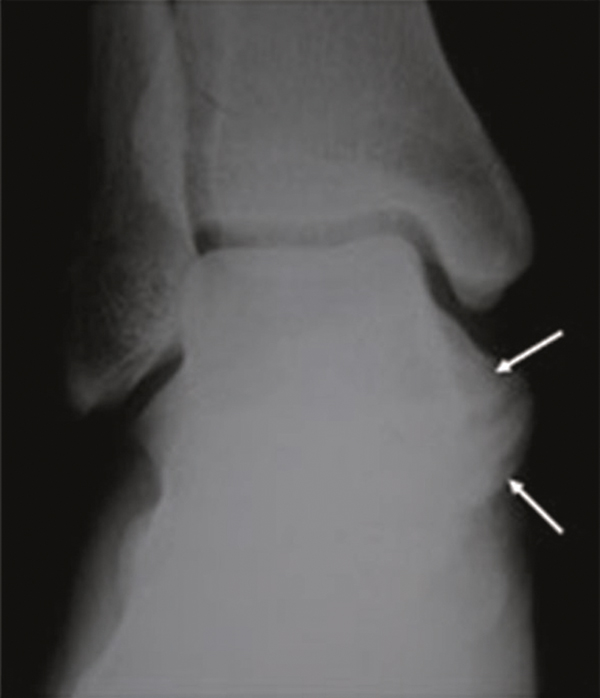
**A preoperative radiograph of the foot shows a bony prominence (arrows) raising from posteromedial of the calcaneus**.

**Figure 2:**
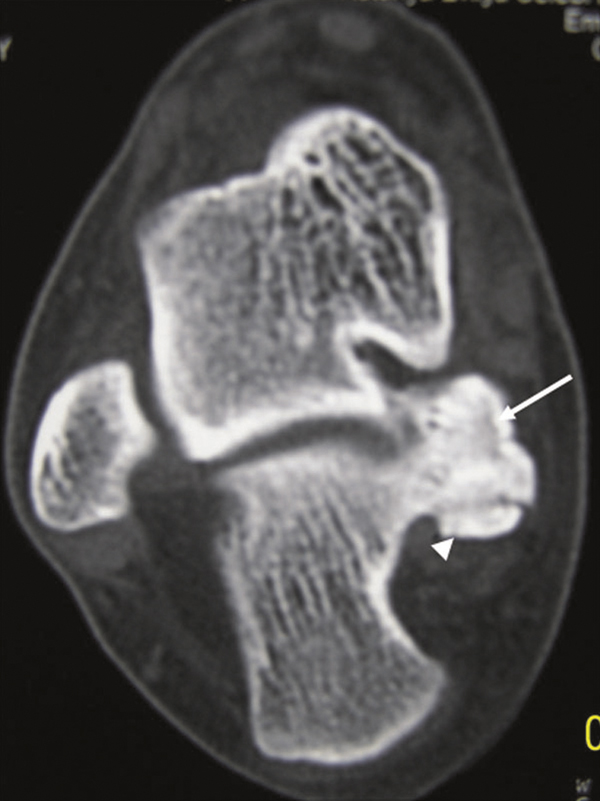
**A preoperative axial CT scan of the foot shows the bone density lesion (arrow) raising from posteromedial of the calcaneus (27 × 23 mm in size, cartilage cap (arrowhead) thickness: 7 mm)**.

**Figure 3:**
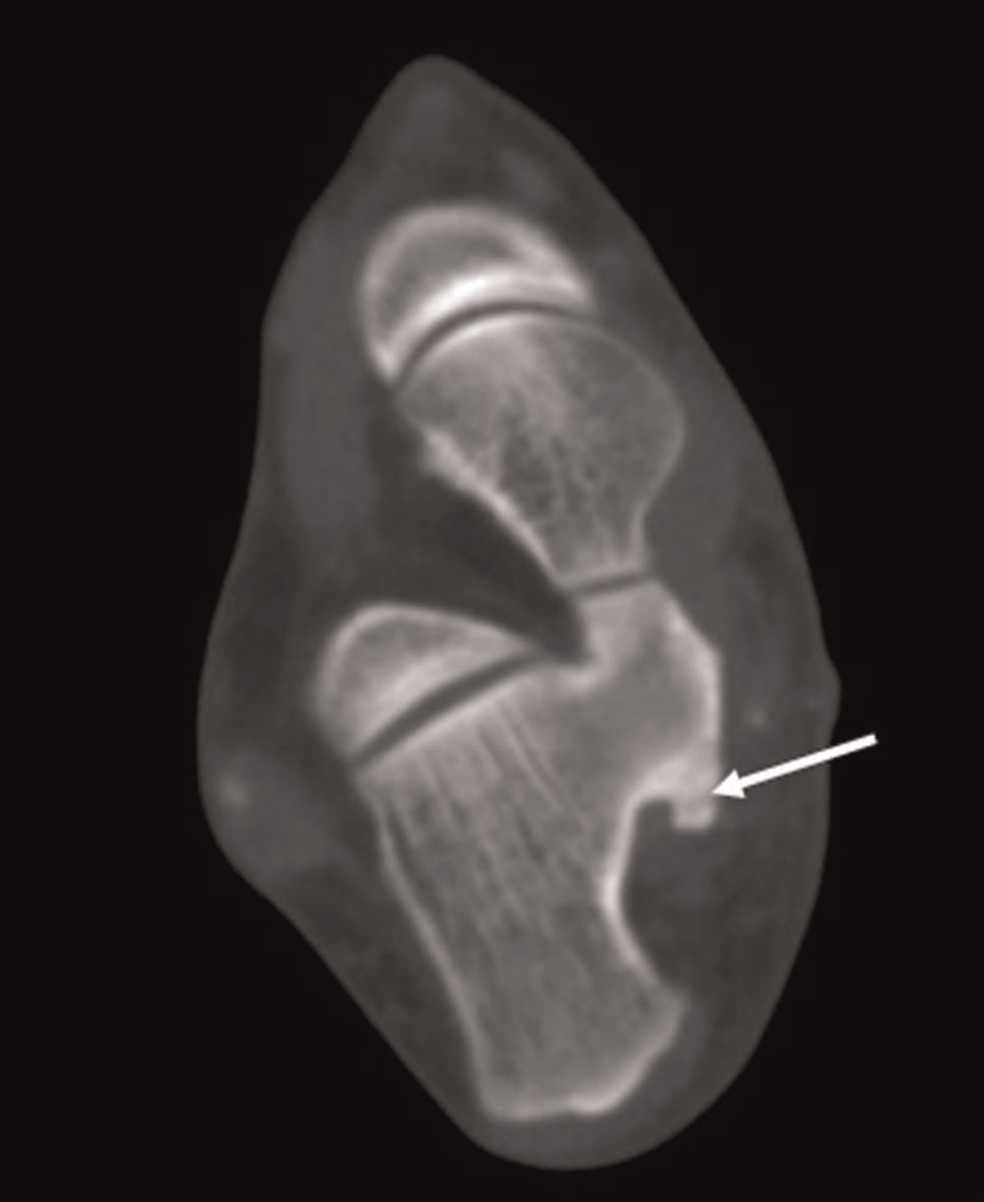
**Axial CT scan taken at the 1-month postoperative of the foot shows an exofitic lesion (arrow) detected posteromedially to the calcaneus (5 × 3 mm in size)**.

**Figure 4:**
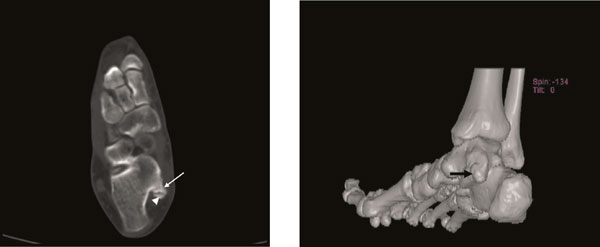
**Axial CT scan (A) and three-dimensional (3D) imaging (B) taken at the 5-month postoperative of the foot shows increase in the lesion sizes (13 × 8 mm) (arrow: osteochondroma, arrowhead: cartilage cap)**.

**Figure 5:**
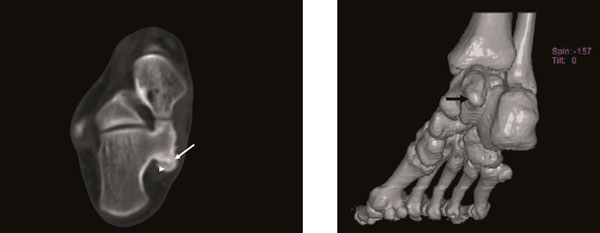
**Axial CT scan (A) and three-dimensional (3D) imaging (B) taken at the 6-month the first-operative of the foot shows increase in the lesion sizes (18 × 12 mm)**. (arrow: osteochondroma, arrowhead: cartilage cap).

**Figure 6:**
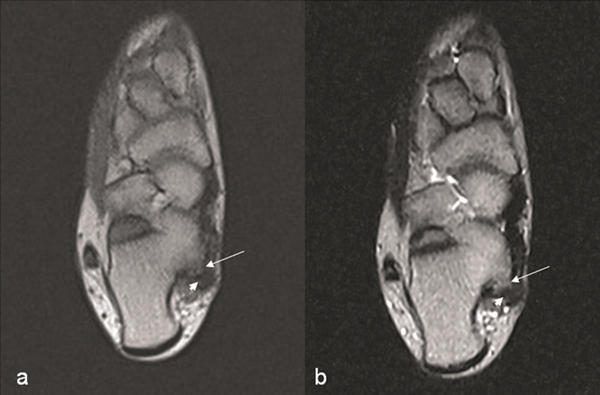
**Axial T1-weighted (A) and T2-weighted (B) MR images taken at the 6-month the first-operative of the foot shows an intermediate signal intensity lesion (arrow) in contact with the calcaneus**. The cartilage cap has shown the low signal intensity in image both T1-weighted and T2-weighted MR (arrowhead: cartilage cap).

**Figure 7:**
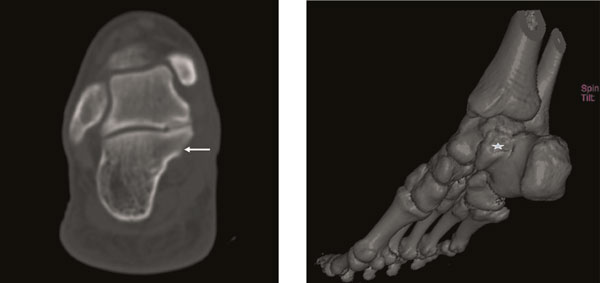
**Axial CT scan (A) and three-dimensional (3D) imaging (B) taken at the 1-month of the second-operation of the foot shows no residual or recurrent lesion (arrow, star)**.

A 25 year-old female patient was admitted to our hospital with painful, stiff mass which was described to be present for nearly one year but increased in size in the last 3 months and became painful recently. In her history no special feature was recorded. In physical examination, about 3 × 2.5 cm stiff, immobile, painful mass placed posteroinferiorly to the medial malleoli was palpated. The mass seemed to be placed on neurovascular structures of the medial ankle. The anterior-posterior (AP) plain x-ray radiography and computed tomography (CT) revealed a bony prominence 27 × 23 mm (cartilage cap thickness: 7 mm) in size raising from posteromedial of the calcaneus and reported to be an osteochondroma (Figure 1, 2). In the operation, it was observed that the lesion raised from the most inferior and posteromedial side of the calcaneus and grew through the surface pushing the soft tissues more posteromedially. The bony mass was excised totally as possible and pathologic examination suggested that it was an osteochondroma. In physical examination on the 15th day the patient was pain free and no new lesion was detected. One month later, the patient again suffered from pain and a smaller and softer lump with tenderness on the operation field was detected. No lesion could detect in plain x-ray radiography. In order to identify the lesion, CT was performed and an exofitic lesion about 5 × 3 mm in size was detected posteromedially to the calcaneus (Figure 3). As it was a small lesion, antinflammatory drugs were given and elastic bandage with ice was applied for the next 15 days but pain persisted and some growing of the lesion again was detected. In the fifth month, a second CT was performed and increase in the lesion sizes (13 × 8 mm) was detected in multiplanar reconstruction and three-dimensional (3D) imaging (Figure 4a and 4b). Cartilage cap thickness was 4 mm. The lesion was reported a benign osteochondroma but as the lesion was persisted on growing and pain increased one month later a third CT study and magnetic resonance (MR) imaging was performed, and some more increase was reported (18 × 12 mm with cartilage cap thickness: 6 mm) (Figure 5a and 5b and Figure 6). As the lesion placed in a relatively atypical region and showed recurrence with pain in a shorter time, malignant transformation was considered. The patient was forwarded to a medical center for musculoskeletal tumours and she was reopereted in this center with an initial diagnosis of chondrosarcoma. The bony prominence was totally excised and pathologic examination again revealed a beningn osteochondroma. The last CT examination that was performed one month later the second operation revealed no residual or recurrent lesion (Figure 7a and 7b). The patient is pain free for 9 months and no new lesion was detected to date.

## Discussion

Osteochondromas are developmental lesions rather than true neoplasms and they may occur solitary or as multiple lesions, associated with the syndrome multiple hereditary exostoses [1]. Osteochondroma is seen in the 2% to 3% of the general population and represents approximately 36% to 41% of the benign bone tumors [7,8]. The hand and feet bones, including the calcaneus comprise only 10% of osteochondromas [3].

The evaluation of osteochondromas can generally help clinical findings, and imaging methods such as plain radiography, ultrasonography, CT, MR imaging and bone scanning [1]-[3].

Osteochondromas are usually asymptomatic, and are seen incidentally on radiography [3]. The most common symptom is a nontender, painless cosmetic deformity seconder to the slowly enlarging exophytic mass. Additional complications that cause symptoms include osseous deformity, fracture, vascular-nerve compression, neurologic sequelae, bursa formation, and malignant transformation [3,7]. Malignant transformation is seen in less than 1% to 2% of patients of solitary osteochondroma [2,9] and in 5%-25% of patients with multiple hereditary exostoses [1,2,10].

Clinical features suspicious for malignant transformation comprise new onset of pain in a previously stable lesion, rapid or new growth, growth after skeletal maturity, and/or large lesions [2,11]. These lesions are usually a low-grade chondrosarcoma or less often a secondary osteosarcoma [12,13]. In our case, the first complaint was pain that reported to be increased recently and a rapid growth of the lesion especially after the first operation.

Although radiography is often diagnostic alone, other imaging modalities may be necessary for surgical planning and to exclude sarcomatous degeneration. The radiographic appearance of this tumor is often diagnostic and reflects its pathologic characteristics. The lesion is composed of native cortical and medullary bone protruding from and continuous with the underlying bone and they appear as sessile or pedunculated [1,2]. However, if there is no extensive mineralization, the thickness of the cartilage cap is usually not well evaluated with radiography [1]. In our case, because of the unusual posteroinferior placement to calcaneus, AP and lateral radiographies did not reveal a satisfactory view.

Ultrasonography can be used in the measurement of the hyaline cartilage cap thickness [14,15]. However, it is an operator-dependent examination with often limited value in obese patients and lack of evaluation of the osseous components of the lesion [1]. Bone scanning is directly correlated with the degree of enchondral bone formation [16,17]. Radionuclide uptake is usually more prominent in young patient. In older patients, it may not demonstrate any uptake. In addition, it has not been useful forevaluating malignant transformation [2].

MR imaging is the best radiologic imaging method evaluating hyaline cartilage cap. It also important for visualizing the effect of the lesion on surrounding structures and shows cortical and medullary continuity between the parent bone and osteochondroma. The high water content in nonmineralized areas of the cartilage cap had intermediate to low signal intensity on T1-weighted images and high signal intensity on T2-weighted images. Mineralized areas in the cartilage cap had low signal intensity on T1 and T2-weighted images [18,19]. However, in young patients with active growth and maturation from normal enchondral ossification in the cartilage cap may be marked heterogeneity both T1-weighted images and T2-weighted images because of the mixture of nonmineralized and mineralize cartilage tissues [1]. In our case, cartilage cap had low signal intensity on T1 and T2-weighted images.

Multiplanar reconstruction and three-dimensional imaging features of CT give important information about determining of these lesions. It allows optimal demonstration of the pathognomonic cortical and medullary continuity of the lesion and parent bone as in our case. Murphey et al [1] believed that very thin sections available with CT are often superior to MR imaging, especially in complex areas of anatomy, in osteochondroma cases. Mineralization in the cartilage cap allows a correct CT measurement as we did in this case. However, it can be very difficult to correctly measure the thickness of a totally nonmineralized cartilage cap because it cannot be easily differentiated from surrounding muscle or bursa. Cartilage cap thickness greater than 1 to 2 cm in adults and 2 to 3 cm in growing children suggests malignant transformation [1,2,20].

The treatment of osteochondromas in the foot is conservative or surgical (excision). Stable, small asymptomatic lesions can be treated conservatively. If the lesion is painful and growing after skeletal maturity, exhibit signs of malignant transformation should be treated surgically. A marginal resection is adequate and shows a low rate of recurrence. Any remaining cartilage cap may result in recurrence, especially in growing lesions [1].

In our case, we thougt that there might be small residue after the first operation because of the difficult placement of the lesion for the surgery. We measured the cartilage cap thickness 4 and 6 mm at the 5th and 6th month of the first operation, respectively, Because of the lesion showed reccurrence and it was painful following the first operation, malignant transformation was clinically considered and the patient was re-operated,. However, histopathological examination did not show malign findings, and there was no recurrence during the 9-months followup.

In conclusion, it should keep in mind that benign osteochondromas can represent symptomatic growth in skeletally mature patients without malignant transformation.

## Abbreviations

AP: Anterior-posterior; CT: Computed tomography; MR: Magnetic resonance.

## Consent

Written informed consent was obtained from the patient for publication of this case report and accompanying images. A copy of the written consent is available for review by the journal's Editor-in-Chief.

## Competing interests

The authors declare that they have no competing interests.

## Authors' contributions

MK: Searching the literature. Detecting the radiological images and writing radiological parts of the text. ST: Searching the literature. Major contributor in writing the manuscript. Writing the case report and discussion section. Operator (1st operation). LS: Anaesthesist of the 1st operation. Contributor in writing (editing the style etc.). VK: Searching the literature. Contributor in writing the case report and discussion section. All authors read and approved the final manuscript.
